# Predicting Motor Outcome of Subthalamic Nucleus Deep Brain Stimulation for Parkinson’s Disease Using Quantitative Susceptibility Mapping and Radiomics: A Pilot Study

**DOI:** 10.3389/fnins.2021.731109

**Published:** 2021-09-07

**Authors:** Yu Liu, Bin Xiao, Chencheng Zhang, Junchen Li, Yijie Lai, Feng Shi, Dinggang Shen, Linbin Wang, Bomin Sun, Yan Li, Zhijia Jin, Hongjiang Wei, Ewart Mark Haacke, Haiyan Zhou, Qian Wang, Dianyou Li, Naying He, Fuhua Yan

**Affiliations:** ^1^Department of Radiology, Ruijin Hospital, Shanghai Jiao Tong University School of Medicine, Shanghai, China; ^2^School of Biomedical Engineering, Institute for Medical Imaging Technology, Shanghai Jiao Tong University, Shanghai, China; ^3^Department of Neurosurgery, Center for Functional Neurosurgery, Ruijin Hospital, Shanghai Jiao Tong University School of Medicine, Shanghai, China; ^4^Department of Radiology, Changshu Hospital Affiliated to Nanjing University of Chinese Medicine, Changshu, China; ^5^Shanghai United Imaging Intelligence Co., Ltd., Shanghai, China; ^6^School of Biomedical Engineering, ShanghaiTech University, Shanghai, China; ^7^Department of Artificial Intelligence, Korea University, Seoul, South Korea; ^8^School of Biomedical Engineering, Med-X Research Institute, Shanghai Jiao Tong University, Shanghai, China; ^9^Department of Radiology, Wayne State University, Detroit, MI, United States; ^10^Department of Neurology, Ruijin Hospital, Shanghai Jiao Tong University School of Medicine, Shanghai, China

**Keywords:** quantitative susceptibility mapping, radiomics, deep brain stimulation, Parkinson’s disease, motor outcome, prediction

## Abstract

**Background:**

Emerging evidence indicates that iron distribution is heterogeneous within the substantia nigra (SN) and it may reflect patient-specific trait of Parkinson’s Disease (PD). We assume it could account for variability in motor outcome of subthalamic nucleus deep brain stimulation (STN-DBS) in PD.

**Objective:**

To investigate whether SN susceptibility features derived from radiomics with machine learning (RA-ML) can predict motor outcome of STN-DBS in PD.

**Methods:**

Thirty-three PD patients underwent bilateral STN-DBS were recruited. The bilateral SN were segmented based on preoperative quantitative susceptibility mapping to extract susceptibility features using RA-ML. MDS-UPDRS III scores were recorded 1–3 days before and 6 months after STN-DBS surgery. Finally, we constructed three predictive models using logistic regression analyses: (1) the RA-ML model based on radiomics features, (2) the RA-ML+LCT (levodopa challenge test) response model which combined radiomics features with preoperative LCT response, (3) the LCT response model alone.

**Results:**

For the predictive performances of global motor outcome, the RA-ML model had 82% accuracy (AUC = 0.85), while the RA-ML+LCT response model had 74% accuracy (AUC = 0.83), and the LCT response model alone had 58% accuracy (AUC = 0.55). For the predictive performance of rigidity outcome, the accuracy of the RA-ML model was 80% (AUC = 0.85), superior to those of the RA-ML+LCT response model (76% accuracy, AUC = 0.82), and the LCT response model alone (58% accuracy, AUC = 0.42).

**Conclusion:**

Our findings demonstrated that SN susceptibility features from radiomics could predict global motor and rigidity outcomes of STN-DBS in PD. This RA-ML predictive model might provide a novel approach to counsel candidates for STN-DBS.

## Introduction

Deep brain stimulation (DBS) targeting the subthalamic nucleus (STN) is a surgical therapy with class I evidence for improving motor symptoms of Parkinson’s disease (PD) (Odekerken et al., 2013). Overall, STN-DBS yields improvements in motor symptoms by approximately 52% when observed in the DBS-on with medication off (DBS-ON/med-OFF) state at 6-month follow-up compared to preoperative med-OFF state (Kleiner-Fisman et al., 2006). However, the global and specific motor outcomes of DBS therapy remain highly variable (Okun et al., 2005) and the underlying mechanisms have not been fully unveiled. Several factors can contribute to the variable outcome after DBS surgery, including candidate selection, target localization, postoperative programming, and medication adjustment (Okun et al., 2005). Most of these key factors can be well-controlled at experienced sites. However, owing to the heterogeneity of patient-specific disease profile, predicting motor response remains challenging. Considering the cost, invasiveness and potential adverse effects of DBS surgery, it is vitally important to predict surgical efficacy accurately when counseling candidates for DBS (Lang and Widner, 2002).

In current clinical settings, satisfactory levodopa challenge test (LCT) responsiveness on motor symptoms has been widely used to select suitable PD candidates for STN-DBS. However, there is currently an ongoing debate regarding whether preoperative LCT response is a reliable predictor or not. Some studies demonstrated its predictive value (Charles et al., 2002) but not the others (Piboolnurak et al., 2007). In addition, the threshold values for the LCT responsiveness have not been standardized for DBS, ranging from 25 to 50% in published surgical series (Vingerhoets et al., 2002; Welter et al., 2002).

PD is a complex movement disorder with different responses to treatment, probably reflecting different pathological and biological contributions (Espay et al., 2017). One of the reasons for the heterogenous therapeutic effects among PD patients could be the heterogeneity underlying PD pathology (Espay et al., 2017). With the advent of novel imaging techniques, it would be necessary to assess pathophysiological process and approach imaging biomarkers to facilitate the development of patient-specific interventions. The neuropathological hallmark of PD is dopaminergic neuronal loss in the iron-rich substantia nigra (SN) (Oakley et al., 2007). The neurodegenerative process is triggered long before the onset of motor disability in PD patients. Since the first report of elevated nigral iron content in a PD patient in 1924. Consistent findings have demonstrated that, over time, excessive iron accumulates in the pigmented neurons of the substantia nigra pars compacta, especially in the form of a neuromelanin (NM)-iron complex (Jellinger et al., 1992; Zecca et al., 1996). Biologically, NM serves as an iron chelator leaving behind a NM-iron complex that activates microglia. Increased ferritin-loaded microglia in the SN initiates and prompts α-synuclein aggregation which links to PD development and progression (Li et al., 2010). The aggregation of α-synuclein and formation of Lewy bodies may lead to a continuing protective response which results in selective neuronal loss in the SN-dopaminergic (DA) system (Zucca et al., 2017). It is believed that one of the most promising neuroimaging biomarker candidates for PD is iron content as assessed with MRI. Several imaging studies observed decreased T2 relaxation times and increased relaxation rates (R2/R2^∗^) in the SN in PD patients compared to healthy controls (Dexter et al., 1989; Martin et al., 2008; Péran et al., 2010; Yan et al., 2018; Ghassaban et al., 2019). An ultra-high field 7 Tesla (7T) MRI study based on T2^∗^-weighted images delineated the nigrosome-1 territory of the SN with increased spatial resolution and new contrast (Lehéricy et al., 2014). Elevated nigral iron content was interpreted as the primary source of imaging changes in PD patients. However, these T2^∗^/R2^∗^ measurements have a non-local component to them owing to the dipole effects of the iron sources (Schweser et al., 2011).

Neuroimaging has revolutionized quantitative iron detection *in vivo* with the advent of quantitative susceptibility mapping (QSM) (Haacke et al., 2015). Today QSM is able to directly represent the source of the iron and, hence, its concentration (He et al., 2015, 2021). Using phase data from multi-echo gradient recalled echo (GRE) sequences, QSM deconvolves the phase to produce a source susceptibility map. Post-mortem studies have validated that QSM provided a reliable and sensitive measurement to deep gray matter iron content (Langkammer et al., 2012). Importantly, QSM opens an avenue to investigate the role of iron deposition in PD pathology (Lotfipour et al., 2012) and clinical outcomes. Furthermore, radiomics with machine learning (RA-ML) analysis based on QSM precisely captures spatial heterogeneity in nigral iron distribution and subsequently has practical clinical applications in PD diagnosis (Li et al., 2019).

Since Langley first proposed the notion of machine learning (ML) in 1996, MRI-based ML for PD research has been widely used in disease classification (Salvatore et al., 2014; Adeli et al., 2016; Huppertz et al., 2016). In addition to the traditional QSM-based analysis of volume and susceptibility in deep gray nuclei between PD and healthy controls (HC) (He et al., 2015), researchers have applied imaging features for further investigation. One recent QSM study observed significant difference of histogram features in several deep gray nuclei between PD patients and HC (Zhang Y. et al., 2020). They also demonstrated that combined model of the 10th percentile of SN (SN_P10_) and the 75th percentile of Putamina (PUT_P75_) successfully distinguished PD patients from HC far outweighing than the mean value (Zhang Y. et al., 2020). In addition to the first-order features of the deep gray nuclei such as mean value and percentile, other radiomics features, for example texture features, were also investigated for PD diagnosis. The texture features reflect the variation and distribution of local tissue properties, which are otherwise not apparently observed by a human reader (Zhang et al., 2012). These features can capture subtle changes within the structure and can be useful for detecting alteration from normal tissue in PD patients. The texture analyses of the SN showed the superiority of QSM to R2^∗^ map in discriminating PD and HC in one study (Li et al., 2019). In order to apply radiomics to patient-specific diagnosis instead of intergroup comparison, the researchers took these radiomics features as prior knowledge and constructed a novel diagnostic model with the approach of ML (Xiao et al., 2019).

Currently, there are no efficient indicators for predicting specific motor outcome, warranting the search for other imaging biomarkers to predict surgical efficacy and screen for suitable candidates. We hypothesized that individual iron distribution in the SN, which may reflect patient-specific disease trait, could potentially account for some variability in motor outcome of STN-DBS surgery. In this study, we introduce a computer-aided PD prognostic framework to develop a RA-ML model to predict motor outcome of STN-DBS when counseling PD candidates for STN-DBS.

## Materials and Methods

### Patients

A total of thirty-three PD patients were prospectively recruited for bilateral STN-DBS surgery from December 2017 to September 2019. This study was approved by the Ethics Committee of Ruijin Hospital Affiliated to Shanghai Jiao Tong University School of Medicine. All patients provided written informed consent prior to recruitment.

The PD patient selection criteria were: (i) clinically diagnosed idiopathic PD based on the United Kingdom PD Society Brain Bank Criteria (Hughes et al., 1992) at the time of surgery; (ii) absence of severe cognitive impairment [Montreal Cognitive Assessment (MoCA) score ≥ 17, or Mini-mental State Examination (MMSE) score ≥ 24] (Hoops et al., 2009); and (iii) no significant neuropsychiatric disorders, such as depression [Beck Depression Inventory (BDI) score ≥ 15], anxiety disorders (e.g., panic disorder and obsessive-compulsive disorder), psychotic disorders (e.g., schizophrenia) or post-traumatic stress disorder; and (iv) no MRI contraindications.

### Clinical Evaluation

Demographic and clinical information including gender, age, disease duration, levodopa equivalent daily dosage (LEDD), and LCT responsiveness were recorded prior to STN-DBS operation. Preoperative Movement Disorder Society-sponsored revision of the Unified Parkinson’s Disease Rating Scale Part III (MDS-UPDRS III) scores were recorded in the off-medication (med-OFF, 12 h discontinuation of levodopa) state. Postoperative MDS-UPDRS III scores and LEDD were obtained in the DBS-ON/med-OFF state at 6-months follow-up. MDS-UPDRS III total scores, corresponding to global motor outcome, were then subdivided to measure improvements in specific motor outcome, including rigidity (item 3.3), bradykinesia (item 3.4–3.8), and tremor (item 3.15–3.17) (Malaga et al., 2021). Motor improvement was calculated as follows,

$$\mathrm{Improvement}\left(\%\right)=\frac{\left(p\,r\,e\,o\,p\,e\,r\,a\,t\,i\,v\,e\,M\,D\,S-U\,P\,D\,R\,S\,I\,I\,I\,s\,c\,o\,r\,e\right)-\left(p\,o\,s\,t\,o\,p\,e\,r\,a\,t\,i\,v\,e\,M\,D\,S\,\text{-}\,U\,P\,D\,R\,S\,I\,I\,I\,s\,c\,o\,r\,e\right)}{p\,r\,e\,o\,p\,e\,r\,a\,t\,i\,v\,e\,M\,D\,S\,\text{-}\,U\,P\,D\,R\,S\,I\,I\,I\,s\,c\,o\,r\,e}\times 100\%$$

Optimal surgical outcome was defined as having achieved no less than 30% improvement, consistent with previously published classification (Rodriguez et al., 2007) and suboptimal outcome was defined as less than 30% improvement. In this work, tremor was not included in the RA-ML analysis given that there were 100% improvement of tremor symptoms in most PD patients (16/33) in the postoperative DBS-ON/med-OFF state.

### MRI Data Acquisition

Prior to STN-DBS operation, all patients were imaged at 3T MRI scanner (Ingenia, Philips Healthcare, Netherlands) with a 15-channel phased head coil. Thin foam pads both under and beside head were used to reduce potential motion artifacts. The imaging parameters were chosen as follows: (1) multi-echo GRE sequence: voxel resolution = 1 × 1 × 1 mm^3^, flip angle (FA) = 12^o^, repetition time (TR) = 25 ms, eight echoes with the first echo times (TE1) = 3.3 ms, echo spacing (ΔTE) = 2.6 ms, sampling bandwidth = 673 Hz/pixel, number of slices = 136, and an acquisition time of 6 min and 16 s; 2) T2-weighted fast spin echo (FSE T2W): TR = 4,000 ms, TE = 106 ms, FA = 90°, voxel resolution = 0.75 × 0.75 × 1.5 mm^3^, bandwidth = 440 Hz/pixel, and an acquisition time of 5 min and 36 s. Fluid attenuated inversion recovery (FLAIR) and diffusion-weighted imaging (DWI) data were also collected to ensure there were no cerebrovascular diseases or space-occupying lesions in the brain. The transverse plane was set to be parallel to the anterior commissure-posterior commissure line.

### Targeting Procedure for STN-DBS

Each patient was defined as a potential candidate for STN-DBS after reaching group consensus among functional neurosurgeons, neurologists, neuropsychologists, and psychiatrists. All patients received bilateral STN-DBS following the standard procedure of screening for DBS eligibility at Ruijin Hospital. The targeting procedure for STN-DBS was performed as described previously (Zhang C. et al., 2020). The electrodes of two manufacturers (model 3387–40, Medtronic, Minneapolis, MN, United States or model L302, PINS, Beijing, China) were used in the recruited PD patients, which had the same electrode size and physical parameters. For each patient, correct electrode placement was verified by postoperative CT imaging merged with preoperative high-resolution FSE T2W images.

### Data Processing and Model Construction

#### Image Pre-processing and Segmentation

QSM reconstruction was performed in MATLAB (MathWorks, Inc., Natick, MA, United States). Original DICOM (Digital Imaging and Communications in Medicine) data from multi-echo GRE sequences were separated into magnitude and phase images. The magnitude images were used to extract brain tissue by using the Brain Extraction Tool (BET) (Smith, 2002) in FSL (FMRIB, University of Oxford, Oxford, United Kingdom). Then, a Laplacian-based algorithm was used to unwrap the raw phase data. Lastly, the susceptibility map was reconstructed through dipole inversion using STAR-QSM (streaking artifacts reduction for QSM) algorithm (Wei et al., 2015).

The regions-of-interest (ROIs) for the SN were manually drawn by two experienced radiologists using SPIN software (SpinTech, Inc., Bingham Farms, MI, United States). They were blinded to clinical details and drew the initial ROIs independently. When tracing the ROIs of SN, the QSM images were zoomed by a factor of four to make sure the objects could be accurately drawn. The ROIs were drawn to cover the bilateral SN on all slices where the SN signal was clearly visible on QSM images. The final ROIs were obtained from the initial ROIs using a semi-automated dynamic programming approach in SPIN software (He et al., 2021). Finally, we combined the final ROIs of all slices unilaterally to obtain the SN volume-of-interest (VOI).

#### Radiomics Feature Extraction

The radiomics feature extraction were performed by PyRadiomics, an open-source platform described in a previous publication (van Griethuysen et al., 2017). In total, 1,328 radiomics features were extracted from the SN VOIs of each patient, including 26 shape features, 252 first-order features, 336 Gray Level Co-occurrence Matrix (GLCM) features, 224 Gray Level Run Length Matrix (GLRLM) features, 224 Gray Level Size Zone Matrix (GLSZM) features, 196 Gray Level Dependence Matrix (GLDM) features, and 70 Neighboring Gray Tone Difference Matrix (NGTDM) features. The shape features were directly extracted from the SN VOIs. The first-order and texture features were extracted from the original QSM image or from the filtered QSM images with one of three specific filter types: (i) Wavelet filtering: Applying high (H) or low (L) pass filters to a 3D image in each of the three dimensions. We performed LLL and HHH to filter QSM images with relative high stability. (ii) LoG: Laplacian of Gaussian filter for highlighting edges. (iii) Gradient: Calculating and returning the gradient magnitude of the image.

#### Feature Selection by Stability

The intraclass correlation coefficient (ICC) was used to quantify the stability degree of each feature between the two raters. ICC is an established tool to assess inter-rater reliability. In this study, ICC estimates was based on an absolute-agreement and two-way random-effects model, which follows the guidelines for choosing the appropriate form of the ICC (Shrout and Fleiss, 1979). Here, ICC (2,1) estimates were calculated for feature values using the Pingouin python package (Vallat, 2018). It is recommended that ICC estimates should be >0.80, which indicates good reliability between the raters. In this study we selected 657 radiomics features whose ICC values were all >0.85, which is a reasonable selection strategy (van Griethuysen et al., 2017). Since ICC is a statistical indicator, it cannot be assumed that each feature with ICC >0.85 will not have a large error for each case. Therefore, a relative error of 10% was used for further feature selection. Relative error is the ratio of an error in a measured quantity to the magnitude of that quantity, which indicates the credibility of the measurement. We preserved 149 features with relative error <0.1, which aimed to select the features with a relatively high degree of credibility of the measurement.

#### Feature Selection by Prior Knowledge

Due to the limited sample size and high-dimensional feature vector, it is important to remove noisy features and avoid potential overfitting problems. As suggested in one study (Amoroso et al., 2018), another validation of the proposed methodology is that the selected features should correspond to the ones whose relationship with the disease has already been established. Therefore, we took prior knowledge into consideration. We assume that the features applied to classify optimal and suboptimal surgical outcomes in PD patients are related to PD pathophysiological changes. Among those 149 features, 10 had the same feature types with those from the feature clusters associated with PD and HC classification in our previous QSM studies (Cheng et al., 2019; Xiao et al., 2019), which were subsequently used for model construction.

#### Logistic Regression With Recursive Feature Elimination

Logistic regression (LR) was employed as a linear model for the classification task (Optimal vs. Suboptimal). After a linear transformation and a nonlinear activation function, all input radiomics features were transformed into classification probability values, ranging from 0 to 1. The value describes the confidence that a PD subject will have optimal surgical outcome. In the training stage, the cost function of LR was given by:

$$m\,i\,n_{w,c}\,\frac{1}{2}\,{||w||}_{p}+\underset{l\,o\,s\,s\,f\,u\,n\,c\,t\,i\,o\,n}{\underbrace{C\sum_{i=1}^{n}log(exp\left(-y_{i}\left(X_{i}^{T}w+b\right)\right)+1}},p\in \left\{1,2\right\}$$

where w and b represent the trainable parameters. X_i_ ∈ R^n^ and y_i_ ∈ {−1, + 1} is the radiomics feature vector and target label representing the *i*th sample. In the loss function, there are two hyperparameters: C ∈ {1,3,5,7,9,13,15} and p ∈ {1,2}, which controls the weight of the loss function and the complexity of the model, respectively.

Combined with LR, recursive feature elimination (RFE) was also employed for feature selection. RFE is a commonly used method of feature selection (Wottschel et al., 2019). And hyperparameters of the features are determined by the algorithm itself, therefore there is little manual intervention. Given that the LR model assigns weights to features, the goal of RFE is to select the most important ones by recursively selecting smaller and smaller sets of features. Firstly, the LR was trained on the initial set of features and the importance of each feature was assessed by its feature coefficient w. Then, the least important features were excluded. This procedure was recursively repeated on the stepwise-changed set until an optimal model was obtained. We set the number of desired features as a hyperparameter N ∈ {5,7,9,11,13}.

#### Development and Validation of the Model

**FIGURE 1:**
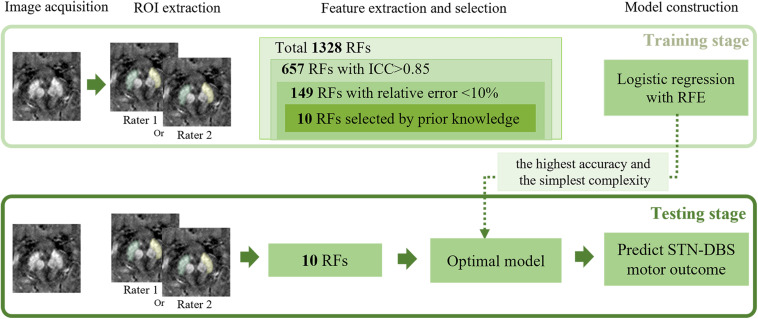
Illustration of the processing pipeline of the RA-ML model construction. RF, radiomics features; ICC, intraclass correlation coefficient; RFE, recursive feature elimination; STN-DBS, subthalamic nucleus deep brain stimulation.

Statistical tests were performed on Scikit-learning software when we developed the LR model. After feature selection by stability and prior knowledge, 10 features were finally used for model construction. All features were normalized by removing the mean and scaling to unit variance. We used Leave-One-Out cross-validator (LOOCV) to test the generalization performance of the model. For each experiment, we randomly selected one sample for testing and the rest samples for training. In the training stage, we selected the best hyperparameter based on the following criteria: selecting the model with the simplest complexity among those with the best training accuracy. Specifically, when accuracies of different models were the same, we chose the one with smaller N ∈ {5,7,9,10},C ∈ {1,3,5,7,9}, and p ∈ {1,2}. A schematic flowchart of the RA-ML processing is shown in Figure 1.

In addition to the RA-ML model based on SN radiomics features, we also constructed the models as follows: (1) the RA-ML+LCT response model, which combined SN radiomic features with preoperative LCT response, (2) the LCT response model alone.

#### Statistical Analysis

Demographic information and clinical details, such as age at surgery, disease duration, preoperative LCT responsiveness, pre- and post- surgical LEDD were assessed in the two groups (Optimal vs. Suboptimal) using an independent sample *t*-test. Patient’s gender was assessed using a Fisher’s exact test. We applied Mann-Whitney *U* test for intergroup comparisons regarding the values of radiomics features. The level of significance was set at *p* < 0.05 for a two-tailed test. The statistical analyses mentioned above were performed in IBM SPSS Statistic Version 26.0 (International Business Machines Corporation, Armonk, NY, United States). Receiver operating characteristic (ROC) curves were constructed and area under the curve (AUC) values were used for assessment of predictive performances. Statistical comparisons of ROC curves among the three models were performed by Delong’s test (DeLong et al., 1988) in MedCalc Statistical Software version 19.7.2 (MedCalc Software Ltd, Ostend, Belgium). The Bonferroni correction was used to correct the error of multiple comparisons in ROC curves. The adjusted *p*-value was set at 0.05/n, where n = times of comparison (The adjusted *p*-value threshold for significance: 0.0167).

## Results

### Participants, Baseline Characteristics, and Follow-Up Outcomes

Demographic and clinical information of all recruited patients are summarized in Table 1. No significant differences of any demographic or clinical characteristics were observed between the two groups (Optimal vs. Suboptimal).

**TABLE 1:** Demographic and clinical characteristics of patients with optimal and suboptimal motor outcome.

	Improvement	N	Age at surgery (Mean ± SD)	Gender (M/F)	LCT response (%)	Disease duration (Years)	Pre-LEDD (mg)	Post-LEDD (mg)
All patients	/	33	60.0 ± 10.1	21/12	48.2 ± 13.6	10.6 ± 4.3	848.1 ± 446.0	433.7 ± 250.6
Global	≥30%	20	59.0 ± 9.6	15/5	49.5 ± 15.0	11.0 ± 4.6	834.5 ± 362.4	389.0 ± 204.3
	<30%	13	61.7 ± 11.1	6/7	46.2 ± 11.5	10.1 ± 4.1	869.0 ± 567.3	498.0 ± 297.7
	*P*	/	0.46	0.14	0.51	0.58	0.83	0.220
Rigidity	≥30%	23	59.1 ± 9.7	15/8	50.7 ± 12.8	10.9 ± 4.3	911.5 ± 494.0	421.9 ± 247.7
	<30%	10	62.1 ± 11.3	6/4	42.3 ± 14.3	9.9 ± 4.4	702.2 ± 277.6	455.2 ± 256.7
	*P*	/	0.45	0.78	0.11	0.54	0.22	0.73
Bradykinesia	≥30%	10	57.4 ± 12.6	8/2	45.4 ± 13.1	12.5 ± 5.3	812.7 ± 402.4	404.0 ± 216.8
	<30%	23	61.2 ± 9.0	13/10	49.4 ± 14.0	9.8 ± 3.6	863.5 ± 471.5	444.1 ± 262.5
	*P*	/	0.33	0.26	0.45	0.10	0.77	0.68

*Data are presented as mean ± standard deviation unless otherwise noted. LCT, levodopa challenge test; LEDD, levodopa equivalent daily dosage.*

### Predictive Performances of the Three Models

**FIGURE 2:**
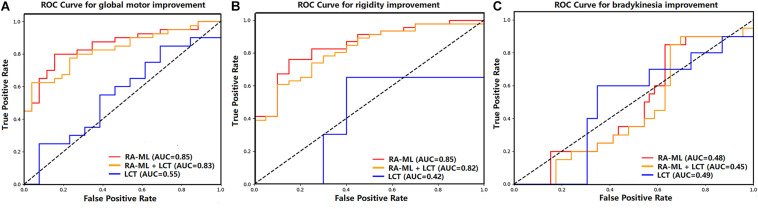
Graph shows receiver operating characteristic curves of the three models predictive of global motor **(A)**, rigidity **(B)**, and bradykinesia **(C)** improvements after STN-DBS.

The predictive performance was evaluated *via* accuracy, balance accuracy, AUC, sensitivity, and specificity (Table 2). Balance accuracy was used to overcome the problem with an imbalanced data set. It normalizes true positive and true negative predictions by the number of the positive and negative samples, which is in accordance with the division of our data into optimal and suboptimal cases, respectively. For the predictive performances of global motor outcome, the RA-ML model had an accuracy of 82% (AUC = 0.85), while the RA-ML+LCT response model had 74% accuracy (AUC = 0.83), and the LCT response model alone had 58% accuracy (AUC = 0.55). For the predictive performance of rigidity outcome, the accuracy of the RA-ML model was 80% (AUC = 0.85), superior to those of the RA-ML+LCT response model (76% accuracy, AUC = 0.82), and the LCT response model alone (58% accuracy, AUC = 0.42). For the predictive performance of bradykinesia outcome, the RA-ML model yielded an accuracy of 50% in comparison with the RA-ML+LCT model (accuracy = 50%) and the LCT response model alone (accuracy = 58%). Figure 2 showed the ROC curves for the three models predictive of global and specific motor outcomes.

**TABLE 2:** Predictive performances of the three models.

Motor outcome	Predictive model	Accuracy	Balance accuracy	AUC	Sensitivity	Specificity
Global	RA-ML	0.82	0.82	0.85	0.80	0.85
	LCT	0.58	0.57	0.55	0.60	0.54
	RA-ML+LCT	0.74	0.75	0.83	0.73	0.77
Rigidity	RA-ML	0.80	0.79	0.85	0.75	0.83
	LCT	0.58	0.58	0.42	0.60	0.57
	RA-ML+LCT	0.76	0.73	0.82	0.65	0.80
Bradykinesia	RA-ML	0.50	0.44	0.48	0.59	0.30
	LCT	0.58	0.58	0.49	0.57	0.60
	RA-ML+LCT	0.50	0.44	0.45	0.59	0.30

*RA-ML model: the model based on SN susceptibility radiomics features.*

*LCT model: the model based on preoperative levodopa challenge test response.*

*RA-ML+LCT model: the model combining SN susceptibility radiomics features with preoperative levodopa challenge test response.*

As shown in Table 3, among the ROC curves of the three models predictive of global motor outcome, the RA-ML model and the RA-ML+LCT response model were significantly higher than the LCT response model alone (*p* = 0.001 and *p* = 0.005, respectively), but the difference was not significant between the RA-ML model and the RA-ML+LCT response model (*p* = 0.359). Multiple ROC comparisons of the three models predictive of rigidity improvement all had significant differences: *p* < 0.001 for the RA-ML and the LCT response model comparison, *p* = 0.011 for the RA-ML and the RA-ML+LCT response model comparison, and *p* = 0.003 for the RA-ML+LCT response and the LCT response model comparison. As for the ROC curves predictive of bradykinesia improvement, there was no significant difference between any two of the models (*p* > 0.0167).

**TABLE 3:** Comparison of ROC curves of the three predictive models.

	RA-ML ∼ LCT	RA-ML+LCT ∼ RA-ML	RA-ML+LCT ∼ LCT
Global	0.001 [0.117, 0.483] ***^a^	0.359 [−0.027, 0.075]	0.005 [0.084, 0.468] **^c^
Rigidity	<0.001 [0.114, 0.425] ***^a^	0.011 [0.007, 0.054] *^b^	0.003 [0.082, 0.396] **^c^
Bradykinesia	0.901 [−0.208, 0.236]	0.303 [−0.022, 0.069]	0.740 [−0.187, 0.263]

*Data are presented as *p*-value [95% confidence interval] unless otherwise noted.*

*Indicates *p* < 0.05; ** indicates *p* < 0.01; *** indicates *p* < 0.001.

*^a^Indicates RA-ML performing better than LCT.*

*^b^Indicates RA-ML performing better than RA-ML+LCT.*

*^c^Indicates RA-ML+LCT performing better than LCT.*

*The adjusted *p*-value threshold for significance after Bonferroni correction is 0.0167.*

### Radiomics Features With Significant Intergroup Differences

**FIGURE 3:**
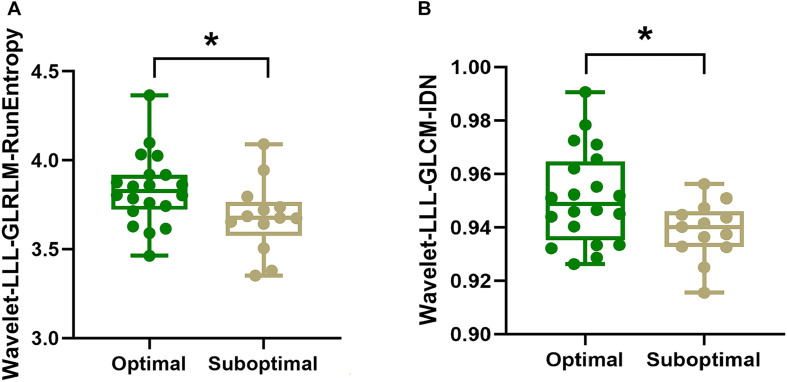
Intergroup comparisons of radiomics features using Mann-Whitney *U* test. As shown in panels **(A,B)**, respectively. The values of Wavelet-LLL-GLRLM-RunEntropy (*p* = 0.036) and Wavelet-LLL-GLCM-IDN (Inverse Difference Normalized) (*p* = 0.039) for the SN are higher in patients with optimal global motor outcome than those who had suboptimal improvement following STN-DBS surgery. In panels **(A,B)**, the box denotes the 25th and 75th percentiles with the horizontal line denoting the median value. * indicates *p* < 0.05.

Among the 10 radiomics features selected for model construction, the values of Wavelet-LLL-GLRLM-RunEntropy (*p* = 0.036) and Wavelet-LLL-GLCM-IDN (Inverse Difference Normalized) (*p* = 0.039) were both significantly higher in patients with optimal global motor outcome as compared to those with suboptimal efficacy (shown in A and B in Figure 3). No significant differences of any other radiomics features were observed between the two groups (Optimal vs. Suboptimal) regarding rigidity or bradykinesia improvements (*p* > 0.05).

## Discussion

To the best of our knowledge, this is the first study to predict motor outcome in PD patients based on individual pre-DBS quantitative images using RA-ML approach. It suggests that RA-ML analysis can capture the subtle differences in iron distribution within the SN structure, which could be identified as a potential imaging biomarker to understand the variability in global motor and rigidity improvements of STN-DBS for PD patients.

### Advantages of the RA-ML Model

In this study, the LCT responsiveness models failed to predict global motor, rigidity and bradykinesia improvements, suggesting that the value of preoperative LCT response for predicting the short-term motor outcome of STN-DBS was limited. However, LCT is widely used to determine whether a patient may benefit from DBS surgery in current clinical settings (Pollak, 2013). These conflicting results may be partly attributed to the enrolled PD patients of different stage in different studies. Importantly, a recent study observed that levodopa medication, rather than DBS itself, had a modulatory effect on basal ganglia activity during finger movement execution (Mueller et al., 2020), which implies different therapeutic mechanisms for levodopa and DBS. Therefore, predicting STN-DBS outcome *via* preoperative LCT responsiveness may be underpowered. Future studies must be carried out for clarification. Importantly, it is reported that levodopa and STN-DBS responses are not congruent (Zaidel et al., 2010). Researchers clarified the methodological shortcomings of previous studies and questioned the validity of using LCT responsiveness as a selection criterion for STN-DBS (Zaidel et al., 2010).

Different from LCT responsiveness, individual nigral iron measurements assessed with QSM data could reflect patient-specific disease trait and potentially help to understand PD prognosis (Bergsland et al., 2019). The predictive performance of the RA-ML model outweighs that of the LCT response model for both global motor and rigidity improvements, which offers a promising alternative to general-applicable LCT responsiveness in counseling PD candidates for STN-DBS. It can be partly explained by the explicit characterization of PD-related pathophysiological changes in the deep gray nuclei by radiomics features, and well-designed modeling between radiomics features and clinical presentation by the ML model. In addition, LCT has its own disadvantages: (1) discontinuation of levodopa for at least 12 h would raise the risk of motor complications; (2) levodopa may prime drug-induced dyskinesias; and (3) LCT is time-consuming.

More recently, imaging studies demonstrated the predictive value of structural and functional connectivity for motor outcome of STN-DBS in PD patients based on diffusion tensor imaging (DTI) (Horn et al., 2017) and resting-state functional MRI (rs-fMRI) (Younce et al., 2020). Smaller ventricular volumes measured on T1 weighted images were associated with more favorable motor outcome in one report (Younce et al., 2019) but not in others (Price et al., 2011). Nevertheless, predicting specific motor outcome based on individual level remains challenging. This study addressed the issues and provided a simple and convenient approach to predict global motor and rigidity outcomes for individual PD patients. Furthermore, since QSM is easily accessible and imaging acquisition is relatively fast, the RA-ML model developed in this study has promising clinical applications to counsel STN-DBS candidates for neurosurgeons’ decision-making.

A previous imaging study reported that the maximum T2-relaxation times (T2) for the SN failed to predict motor outcome of STN-DBS (Lönnfors-Weitzel et al., 2016). This conflicting finding with our work could be partly explained by the fact that change in water content can also change T2 acting as a confounder, but it does not affect the susceptibility measurements. Hence QSM outperforms T2 in measuring nigral iron content and potentially reflecting patient-specific disease trait. In addition, they calculated the absolute instead of the relative improvement in MDS-UPDRS III score. The relative improvement may be preferred to measure surgical efficacy since the absolute improvement calculation only indicates the magnitude of the improvement (Zaidel et al., 2010).

### Interpretability of the RA-ML Model

The level of interpretability is about understanding how the RA-ML model makes decisions, which could help to comprehend the outcome based on the key radiomics features. Among the 10 features selected for model construction, the values of Wavelet-LLL-GLRLM-RunEntropy and Wavelet-LLL-GLCM-IDN for the SN were both significantly higher in patients with optimal global motor outcome as compared to those with suboptimal efficacy. GLRLM-RunEntropy in each VOI measures the randomness of run length and gray distribution. GLCM-IDN is another measure of the local homogeneity of an image. For both these radiomics features, a lower value indicates more homogeneity in the texture patterns, suggesting that those PD patients exhibiting more uniform iron signal with decreased iron heterogeneity may respond worse to STN-DBS treatment.

This finding is comparable to the iron heterogeneity changes underlying PD pathology. Different from the iron-rich SN pars reticulata (SNpr), the lateral-ventral part of the SNpc is packed with melanized dopaminergic neurons with less iron content (Damier et al., 1999). Imaging studies have demonstrated that iron increases predominantly occurred in the lateral-ventral SNpc (Langley et al., 2017; He et al., 2020) in PD; this is in line with the pathological findings (Damier et al., 1999). Therefore, this region-specific high iron accumulation changes the spatial patterns of nigral iron content which could lead to decreased heterogeneity of iron distribution in PD patients. Importantly, susceptibility measurements using RA-ML could capture this subtle difference in spatial iron distribution within the SN structure.

Taken together, this study suggests that the heterogeneity of spatial iron distribution in the SN may be pertinent to patient-specific pathological patterns, which could help to understand the variability of motor outcome in PD patients after STN-DBS.

### Prediction of Specific Motor Outcome

This study conducted RA-ML analysis of specific motor outcome which is not available in most previous studies (Price et al., 2011; Lönnfors-Weitzel et al., 2016; Horn et al., 2017; Younce et al., 2019, 2020). Cardinal motor symptoms in PD patients include rigidity, bradykinesia, and tremor. At least 89% of PD patients suffered from rigidity causing stiffness or inflexibility in muscles (Mutch et al., 1986). In this study, the predictive performance of the RA-ML model was superior to the LCT response model regarding rigidity improvement. The mechanisms underlying the high-precision performance of the RA-ML model predictive of rigidity improvement are not yet fully understood; however, this finding may suggest that susceptibility measurements of nigral iron accumulation could help to understand the variability in rigidity outcome of STN-DBS for PD patients. Bradykinesia is one of the early signs of PD characterized by a reduced ability to move. However, none of the three models in this study were able to predict bradykinesia improvement. This may partly be explained by the fact that STN-DBS treatment affected each motor symptom *via* different mechanisms (Temperli et al., 2003).

### Limitations

There are several limitations to this study. Firstly, the sample size is relatively small, which might be the reason for the poor performance of bradykinesia prediction. In order to prevent the potential overfitting problems resulting from small sample size, we used two sets of VOIs, took prior knowledge into consideration and applied recursive feature elimination to preserve those features with the topmost importance. Clearly, future investigation in a larger cohort is needed. Secondly, The RA-ML analyses of specific motor symptom excluded tremor given that the absence of tremor symptoms in most PD patients (16/33) postoperatively. In order to investigate tremor prediction, more PD patients with suboptimal tremor improvement should be collected in the future. In addition to improving motor symptoms of patients with PD, STN-DBS aims to treat motor complications, such as increasing on-time without dyskinesia and improving motor fluctuation during off periods. Owing to the relatively low presence of levodopa induced dyskinesia (LID) as a result of lower medication dose, the RA-ML analysis of related motor complications was not performed. Future studies with a much larger sample size including more PD patients with motor complications is needed to address this issue. Finally, it would be of interest to perform longer term follow-up analyses, incorporating continued disease progression to explore the fading of DBS efficacy after the surgical honeymoon period (Fasano and Merello, 2020).

## Conclusion

Our study demonstrated that individual SN susceptibility features derived from radiomics can predict global motor and rigidity outcomes of STN-DBS in PD patients. This RA-ML predictive model may provide a novel and practical approach to counsel suitable candidates for neurosurgeons’ decision-making. Our research suggests that individual spatial heterogeneity of nigral iron distribution may be pertinent to patient-specific pathological patterns, which could help to understand the variability of STN-DBS motor benefits.

## Data Availability Statement

The raw data supporting the conclusions of this article will be made available by the authors, without undue reservation.

## Ethics Statement

The studies involving human participants were reviewed and approved by the Ethics Committee of Ruijin Hospital affiliated to Shanghai Jiao Tong University School of Medicine. The patients/participants provided their written informed consent to participate in this study.

## Author Contributions

YLiu, BX, and CZ: writing–original draft, writing–review and editing, investigation, data curation, and formal analysis. JL, YLai, FS, DS, LW, BS, YLi, ZJ, HW, EH, and HZ: data curation, formal analysis, and writing–review and editing. QW, DL, and NH: conceptualization, supervision, funding acquisition, resources, and writing–review and editing. FY: supervision, funding acquisition, resources, and writing–review and editing. All authors contributed to the article and approved the submitted version.

## Conflict of Interest

FS and DS were employed by the Shanghai United Imaging Intelligence Co., Ltd., Shanghai, China. The company has no role in designing, performing the surveillances, analyzing, and interpreting the data. The remaining authors declare that the research was conducted in the absence of any commercial or financial relationships that could be construed as a potential conflict of interest.

## Publisher’s Note

All claims expressed in this article are solely those of the authors and do not necessarily represent those of their affiliated organizations, or those of the publisher, the editors and the reviewers. Any product that may be evaluated in this article, or claim that may be made by its manufacturer, is not guaranteed or endorsed by the publisher.
